# Comparison of popular enrichment methods for untargeted in vitro metabolomics

**DOI:** 10.1007/s11306-025-02309-0

**Published:** 2025-07-27

**Authors:** Yannik Schermer, Frederic Wagner, Simone Stegmüller, Elke Richling

**Affiliations:** Department of Chemistry, RPTU University Kaiserslautern-Landau, Kaiserslautern, Germany

## Abstract

**Introduction:**

Untargeted metabolomics is a popular method by which researchers measure a large portion of the metabolites present in a biological system at once. This approach usually results in complex data sets containing tens to hundreds of thousands of observations which require sophisticated data analysis workflows. To help with the functional interpretation of the data, researchers often rely on enrichment analysis. However, little advice is available on what method to use, and, to the best of our knowledge, there is no comparison of popular approaches available for in vitro data with a focus on toxicological and pharmacological testing.

**Objectives:**

In this study, we compared three popular enrichment analysis approaches—Metabolite Set Enrichment Analysis (MSEA), Mummichog and Over Representation Analysis (ORA)—with data obtained by treating Hep-G2 cells with 11 compounds with five different mechanisms of action. We compared the results and assessed the consistency of the individual methods as well as their correctness.

**Methods:**

Hep-G2 cells were treated with subtoxic concentrations of 11 test compounds. After preparation, samples were measured on an Elute UHPLC coupled to a timsTOF Pro (both Bruker). Spectra were processed in MetaboScape (Bruker) and annotated using spectral library search. Datasets were further processed using R and enrichment analysis was performed in MetaboAnalyst.

**Results:**

Overall, we observed a low to moderate similarity between different enrichment methods with the highest similarity between MSEA and Mummichog. Further, Mummichog outperformed both MSEA and ORA in terms of consistency and correctness.

**Conclusion:**

In our comparison, Mummichog showed the best performance for in vitro untargeted metabolomics data.

**Supplementary Information:**

The online version contains supplementary material available at 10.1007/s11306-025-02309-0.

## Introduction

In recent years, metabolomics has gained increasing interest in the toxicological and pharmacological community (Mussap et al., 2020; Olesti et al., 2021; Ramirez-Hincapie et al., 2023; Viant et al., 2024). Metabolomics can be broadly classified into targeted and untargeted experiments. Targeted experiments aim to quantitate a relatively small number of metabolites while untargeted studies measure as many known and unknown features as possible (Liu & Locasale, 2017). Arguably the greatest strength of metabolomics, especially of untargeted approaches, may sometimes turn into a curse: Its wealth of information. While targeted studies usually focus on tens or hundreds of metabolites, untargeted approaches may yield thousands of features, often leaving researchers puzzled about the underlying meaning of the data. This can also pose a problem for toxicological and pharmacological studies where one tries to understand a compound’s mechanism of action (MOA) by analyzing the metabolic changes the compound causes. One way of turning metabolic profiles of unknown compounds into mechanistic insight is to compare them with profiles of compounds with known MOAs. A prominent example is MetaMap® Tox by BASF SE and its subsidiaries (Kamp et al., 2012). While the underlying concept of this elegant approach has recently been proven to work for in vivo experiments in an impressive ring trial (Viant et al., 2024), concluding MOAs from metabolic profiles alone requires an extensive in-house database. In the case of MetaMap® Tox this database contains the metabolic profiles of around 1000 compounds (Kamp et al., 2012; Van Ravenzwaay et al., 2024).

A faster and cheaper way to put metabolic changes into a biological context and, in the best case deduce mechanistic information, is enrichment analysis. This approach tries to identify pathways or metabolite sets of interest and not individual compounds (Lu et al., 2023). In other words, they show ‘the whole forest’, rather than the individual trees. Enrichment analysis itself is not a new concept. In fact, most approaches were originally developed for other ‘omics platforms (Al-Shahrour et al., 2004; Goeman & Bühlmann, 2007; Subramanian et al., 2005), and many have been adapted for metabolomics (Pang et al., 2024). Probably the most popular ones are over-representation analysis (ORA) (Al-Shahrour et al., 2004; Goeman & Bühlmann, 2007), metabolite set enrichment analysis (MSEA), adapted from gene set enrichment analysis (GSEA) (Subramanian et al., 2005) and Mummichog (Li et al., 2013) because they are implemented in MetaboAnalyst, a widely used web application for metabolomics studies (Pang et al., 2024). The methods and their underlying statistics are explained in detail in a recent publication by Lu et al. (2023).

Due to their different statistical approaches, it can be expected that these methods may yield substantially different results. Indeed, Lu et al. (2023) found considerable discrepancies when they benchmarked popular approaches for enrichment analysis using simulated data as well as published in vivo data sets from patients with coronavirus disease 2019 (COVID-19) and inflammatory bowel disease (IBD). However, what is lacking to this day, to the best of our knowledge, is a systematic comparison of the methods for in vitro data, especially in the context of pharmacological and toxicological testing. To fill this knowledge gap, we treated Hep-G2 cells, a hepatoblastoma cell line widely used in toxicology and pharmacology (Stanley & Wolf, 2022) with a panel of 11 compounds with five different MOAs (Table 1). We covered a range of different MOAs with compounds acting on enzymes of glycolysis, the pyrimidine metabolism and the cholesterol biosynthesis as well as compounds that form reactive oxygen species (ROS) and compounds that interfere with the electron transport chain. The goal of this work was to answer three main questions: (i) How is the consistency among different methods and (ii) among compounds with a similar MOA and, (iii) probably the hardest question to answer, which method yields the most correct pathways. For enrichment methods to be able to meaningfully contribute to mechanistic elucidations of new compounds the results they produce must be correct.

**Table 1:** Mechanism of Action of the compounds used in this work

Compound	Mechanism of Action	Reference
2-Deoxyglucose	Inhibition of **hexokinase**, a key enzyme in glycolysis	(Pelicano et al., 2006)
3-Bromopyruvic acid	Inhibition of **hexokinase**, a key enzyme in glycolysis, also acts as an alkylating agent	(Pelicano et al., 2006)
5-Fluorouracil	Inhibition of **thymidylate synthase**, an enzyme that converts dUMP to dTMP, also causes DNA damage	(Longley et al., 2003)
Antimycin A	Inhibition of complex III of the **electron transport chain**	(Stanford & Taylor-Clark, 2018)
FCCP	Mitochondrial uncoupler that interferes with the **electron transport chain,** indirect inhibition of ATP-synthase in the electron transport chain	(Kenwood et al., 2014)
Menadione	**ROS** generation	(Loor et al., 2010)
Metrizamide	Inhibition of **hexokinase**, a key enzyme in glycolysis	(Bertoni, 1982)
Mevastatin	Inhibition of **HMG-CoA reductase**, a key enzyme in cholesterol biosynthesis	(Endo et al., 1976)
Phenanthrene-9,10-dione	**ROS** generation	(M. Yang et al., 2018)
Simvastatin	Inhibition of **HMG-CoA reductase**, a key enzyme in cholesterol biosynthesis	(Knox et al., 2024)
Trifluorothymidine	Inhibition of **thymidylate synthase**, an enzyme that converts dUMP to dTMP. Also causes DNA damage	(Knox et al., 2024)

## Materials and methods

### Compound selection

A literature search was performed, and compounds were selected to cover a broad range of pharmacologically and toxicologically relevant MOA. Structures are given in Supplement S1.

### Materials and chemicals

Compounds were obtained from various sources (BLD Pharmatech, Germany, Sigma-Aldrich Chemie, Germany and Thermo Fisher Scientific, USA). All compounds had a purity of ≥ 95% and identity was confirmed via ^1^H NMR. Relevant solvents and salts were mass spectrometry (MS) grade.

### Cell culture conditions

Hep-G2 (RRID:CVCL_0027) cells were cultivated in Gibco RPMI 1640 medium (Thermo Fisher Scientific, USA) with 10% Gibco fetal bovine serum (FBS) (Thermo Fisher Scientific, USA) and 1% Gibco penicillin streptomycin (PS) (Thermo Fisher Scientific, USA) at 37 °C and 5% CO_2_ and split with a 1:5 or 1:4 ratio when approximately 80% confluency was reached using Gibco trypsin–EDTA (Thermo Fisher Scientific, USA).

### Dose-finding

For dose finding via resazurin reduction assay, compounds were dissolved in dimethyl sulfoxide (DMSO) (Merck, Germany), except for 3-bromopyruvic acid which was dissolved in double distilled water to prevent decay. Various dilutions were prepared. Dilutions were mixed with RPMI 1640 medium, supplemented with 5% FBS and 1% PS. Final DMSO concentration was 0.5%. A suspension containing 60,000 cells was put in each well of a 96 well plate and incubated for approximately 24 h under cell culture conditions. Subsequently, cells were incubated with compound mixture for 2 h. Vehicles served as negative controls while 0.1% sodium dodecyl sulfate (SDS) served as a positive control. After 2 h cells were washed with 200 µl phosphate-buffered saline (PBS) (37 °C). RPMI 1640 medium containing 1% PS and 10% resazurin solution was added and cells were incubated for an additional hour. Finally, fluorescence was measured (37 °C, 544 nm excitation, 590 nm emission) using a BioTek Synergy H1 microplate reader (Agilent, USA).

Dose–response modelling was done in R version 4.4.2 (R Core Team, 2024) using the drc package (Ritz et al., 2015). Blanks were subtracted, and values were normalized to their vehicle control and the concentration which causes a 10% decrease in viability (i.e., the IC_10_) was derived.

### Cell treatment and sample preparation for endometabolome analysis

The protocol was modified after Bi et al. (2013). Initially, four million cells were seeded in 60 mm dishes and cells were incubated for approximately 24 h under cell culture conditions. Dishes containing 6 ml PBS without cells served as a process blank. Compounds were prepared as described in the section above. Final concentrations in the medium were the calculated IC_10_ values for the respective compound (Supplement S2). After 24 h, medium was removed, and cells were incubated with 6 ml medium containing compound or vehicle control for 2 h at 37 °C and 5% CO_2_. Treatment was carried out in quadruplicates for each compound, vehicle control and process blank. To avoid systematic errors, a new vehicle control and process blank was done for each batch. Medium was removed and cells were washed with 2 ml PBS (37 °C), twice. Metabolism was quenched by adding 400 µl double distilled water cooled to 4 °C containing protease inhibitor (Sigma-Aldrich Chemie, Germany) quickly followed by liquid nitrogen. Cells were transferred to 1.5 ml reaction tubes and immediately flash frozen in liquid nitrogen. Cells were lysed via two rounds of freeze and thaw with 5 min at 37 °C under heavy shaking followed by 1 min in liquid nitrogen and two subsequent rounds of sonication on ice for 10 s with 10 s pause in between. After, 300 µl lysate was transferred to fresh 1.5 ml reaction tubes and 900 µl chilled methanol (Merck, Germany) (-20 °C) was added. Samples were incubated for 10 min at 4 °C under heavy shaking and subsequently incubated for 1 h at − 20 °C to aid protein precipitation. Samples were centrifuged at 14,000 × g and 4 °C for 15 min and 1 ml of supernatant was transferred to a fresh reaction tube. Solvent was evaporated under a gentle nitrogen stream at room temperature. Dried residues were stored at − 80 °C. Prior to the measurements, 100 µl methanol/acetonitrile (VWR Avantor, USA)/water (2:2:1) was added to the residues. They were dissolved by incubating for 10 min at 10 °C under heavy shaking followed by 10 min sonication on ice in a sonication bath. Samples were centrifuged again at 14,000 × g and 4 °C for 10 min and transferred to a vial for Ultra-High Performance Liquid Chromatography (UHPLC) MS analysis. Lysates of untreated Hep-G2 cells served as quality controls (QC).

### Liquid chromatography and mass spectrometry

UHPLC-MS measurements were carried out using an Elute UHPLC pump (Bruker Daltonik, Germany) coupled to a timsTOF Pro (Bruker Daltonik, Germany). Each sample was analyzed in reversed phase (RP) and hydrophilic interaction chromatography (HILIC) positive and negative mode. For RP measurements, a ZORBAX Eclipse Plus C18 column (Agilent, USA, 2.1 × 50 mm, 1.8 µm) was used. HILIC measurements were carried out using an ACQUITY UPLC BEH Amide column (Waters, USA, 2.1 × 50, 1.7 µm). Ion mobility and mass accuracy were calibrated according to the instrument manufacturer prior to each measurement series. Additionally, mass accuracy was calibrated during each measurement using a 10 mM sodium formate (Thermo Fisher Scientific, USA) solution in water/isopropyl alcohol (Honeywell Specialty Chemicals Seelze, Germany) (1:1). The MS was operated in Parallel Accumulation–Serial Fragmentation (PASEF) mode. The mass range was set to *m/z* 20–1,300 with 1/*K*_0_ 0.45–1.45 Vs/cm^2^ and 100 ms ramp time. End plate offset of the ESI source was 500 V with a capillary voltage of 4500 V or 3600 V for positive and negative ion mode, respectively. Nebulizer was set to 2.2 bar and dry gas flow was 9 l/min. Dry temperature was 220 °C. PASEF settings were as follows: Absolute collision energies were 20 eV to 50 eV. Active exclusion was enabled, and exclusion time was 6.0 s with the option to reconsider the precursor if the intensity doubled enabled. The number of PASEF scans was two and the intensity threshold was 100. Each sample was injected three times. QC samples were injected before, during and after sample measurements to allow for within-batch correction. Exact LC and MS parameters are provided in Supplements S3–S5.

### Data processing, treatment and analysis

LC–MS spectral files were processed in Compass MetaboScape 2021b (Bruker Daltonik, Germany). Processing parameters are available (Supplement S6). For MSEA and ORA, metabolites were identified via spectral library search. Three proprietary and one open database were searched hierarchically in the following order: 1. Bruker MetaboBASE Personal Library 3.0 (215,367 spectra of 100,679 compounds), 2. Bruker NIST 2020 MSMS Spectral Library (1,021,914 spectra of 27,840 compounds), 3. MSDial V17 ESI( ±)-MS/MS from authentic standards (obtained from https://systemsomicslab.github.io/compms/msdial/main.html MSP in August 2022, 324,191 spectra of 21,126 compounds and 3848 spectra of 2553 compounds for the positive and negative mode libraries, respectively), 4. Bruker HMDB Metabolite Library 2.0 (6022 spectra of 824 compounds). Only annotations validated by MS/MS spectra were allowed and MS/MS spectra were filtered by precursor *m/z*. The following maximum tolerances were set: *m/z* 5.0 ppm, mSigma 120, MS/MS score 200 and collision cross section (CCS) 5.0%. In MetaboScape, mSigma is a score to describe the deviation between the expected and the measured isotope pattern (the lower the better) and the MS/MS score is an arbitrary number between 1000 (perfect match between the compound MS/MS spectrum and the library MS/MS spectrum) and 0 (no match between the compound MS/MS spectrum and the library MS/MS spectrum). Annotated feature tables were exported to CSV files and further processed in R version 4.4.2 (R Core Team, 2024) using several libraries (Korkmaz et al., 2024; Robinson et al., 2024; Wickham et al., 2019). Briefly, features were filtered out if they were also present in blank samples (i.e., their maximum intensity in samples was less than or equal to three times the maximum intensity of that feature in blank samples), if they showed poor reproducibility (i.e., their coefficient of variation in quality control samples was larger than 0.2) or if they were not found reliably across samples (i.e., if they were not found in at least 75% of the samples of either treatment or control group). Missing values for remaining features were replaced with 1/5 the minimum measured intensity for that feature, intensities were normalized sample-wise by dividing them by the sample median (Ramirez et al., 2018). Technical replicates were combined by calculating the mean for each feature. KEGG (Kanehisa et al., 2025) identifiers were mapped to compound names via compound ID conversion in MetaboAnalyst 6.0 (Pang et al., 2024) and manual search. Feature tables for the same compound obtained with different separation methods and polarities were combined and intensities were log_2_-transformed. Additional method-specific preparation steps were performed due to different input requirements. For ORA, *p*-values were calculated using two-sided Welch’s *t*-test. Metabolites were considered significantly changed if *p* ≤ 0.05 and their change was at least 1.5-fold. For each compound, a reference metabolome containing all annotated metabolites was provided in the pathway analysis to account for method bias (Wieder et al., 2021). As the MSEA module on MetaboAnalyst 6.0 does not allow for duplicates, duplicate metabolites were removed. For this, *t*-scores were calculated using log_2_-transformed intensities. Only the duplicate with the highest absolute *t*-score was kept and others were removed. For Mummichog, *p*-values and *t*-scores were calculated based on log_2_-transformed intensities. The input consisted of *m/z*, *p*-values and *t*-scores.

Enrichment analysis was done using MetaboAnalyst 6.0 (Pang et al., 2024). Additional parameters are given in Supplement S7. Further data analysis was carried out in R version 4.4.2 using several libraries (Brand, 2024; Ching, 2024; Clarke et al., 2023; Kuhn et al., 2022; Pedersen, 2024; Pedersen & Crameri, 2023; R Core Team, 2024; Tenenbaum & Maintainer, 2024; Wickham, 2023; Wickham et al., 2019, 2023). Metabolites covered by each method and their associated pathways are given as supplemental files (Supplementary file 2).

### Measures of similarity

Similarities for different methods and compounds were assessed using two approaches. Pairwise Jaccard indices *J(A,B)* were calculated with

$$J\left( {A,B} \right) = \frac{A \cap B}{{A \cup B}} $$

where *A* and *B* are significantly enriched pathways (*p* ≤ 0.05) for the two methods or compounds. Additionally, Spearman’s correlation coefficient ρ was calculated for each combination of methods and compounds.

### Assessment of the correctness of the results for different enrichment methods

To assess the correctness of the results, the lists of enriched pathways were compared to a list of expected pathways for each compound. To obtain the list of expected pathways, the KEGG database (Kanehisa et al., 2025) was searched for the primary molecular target of each applied compound (Table 1). Pathways associated with each target were downloaded and filtered to only contain those present in the model for MSEA, ORA or Mummichog (Supplement S8). As a measure of correctness, the share of expected pathways correctly identified amongst the top n (1 ≤ n ≤ 20) enriched pathways was calculated for each enrichment method and compound. For example, 3-bromopyruvic acid’s primary target is hexokinase. This enzyme is associated with six pathways present in the model for MSEA, ORA or Mummichog. Of these six pathways, Mummichog was able to identify two if the top ten most enriched pathways are considered, corresponding to 2/6 or approximately 33% of the expected pathways correctly identified.

## Results

**Fig. 1:**
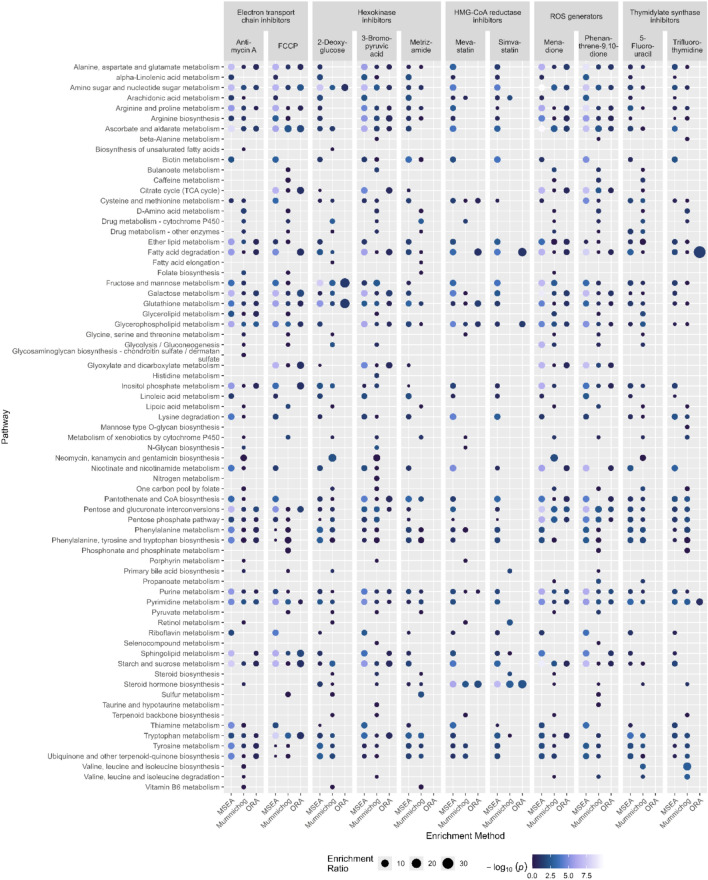
Overview of the pathways obtained with different enrichment analysis methods, faceted by compound and mechanism of action

Data sets obtained for each compound with the three enrichment analysis methods ORA, MSEA, and Mummichog were analyzed. An overview of the results is given in Fig. 1. In the following sections, the three main research questions are answered. (i) Whether the results from different methods are comparable (ii) which method delivers consistent results under similar conditions (i.e., when cells are treated with compounds with a similar MOA) and (iii) which method delivers correct results.

### Are the results from different methods comparable?

**Fig. 2:**
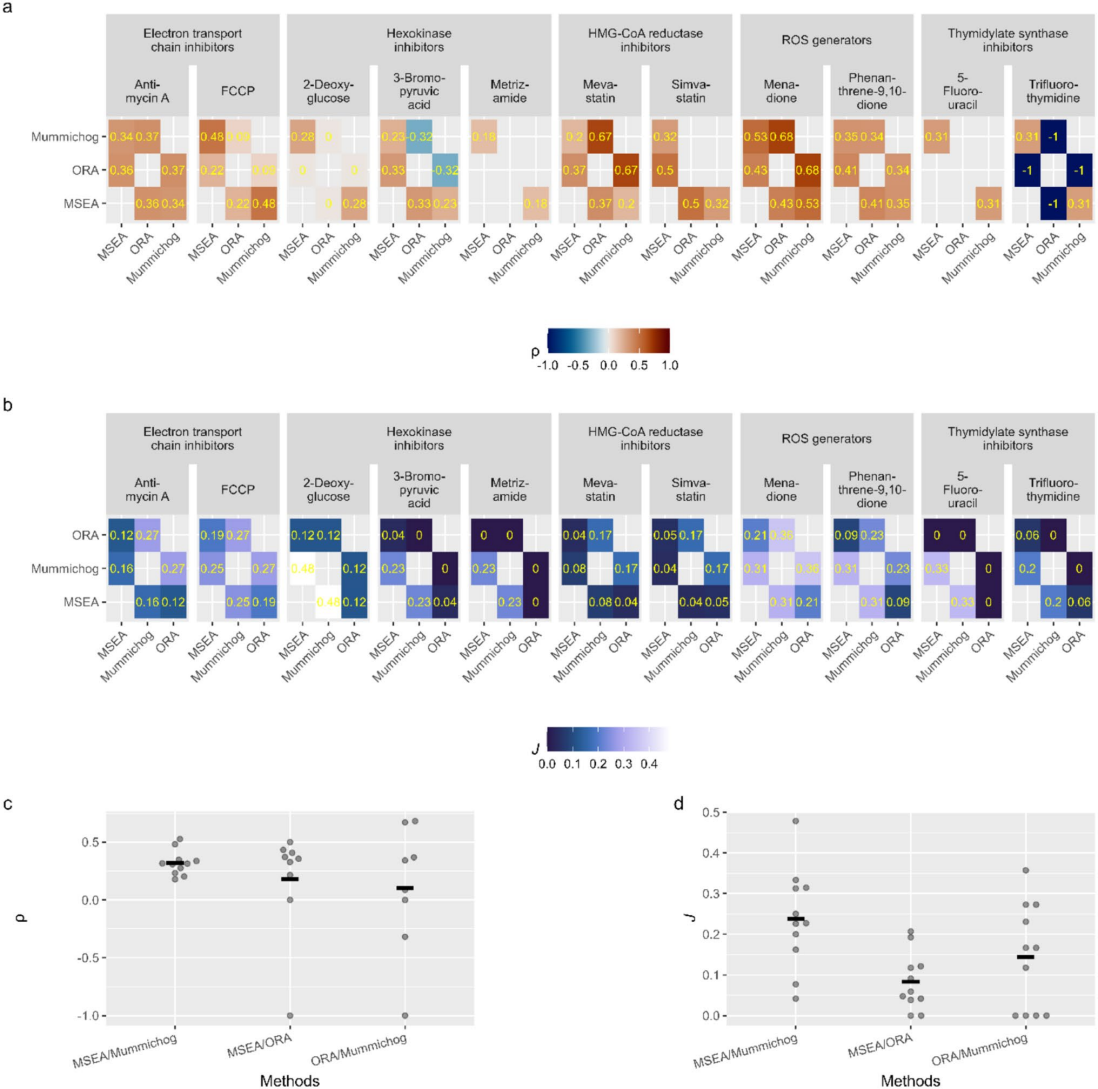
Comparison of the results obtained with different enrichment methods for the same compound. **a** shows the calculated correlation coefficients (Spearman’s ρ), **b** the Jaccard indices *J.* **c** and **d** show the values from **a** and **b** in an alternative way. Black vertical lines represent the means

To assess the first research question, pairwise correlation coefficients of the negative decadic logarithm of the *p*-values associated with each pathway for cells treated with a certain compound and data analyzed with different enrichment analysis methods were calculated. Further, pairwise Jaccard indices were calculated for significantly enriched (*p* ≤ 0.05) pathways as a second measure of similarity. The results are summarized in Fig. 2. Overall, the different methods showed a low to moderate similarity. For both metrics, the highest mean similarity was observed between MSEA and Mummichog (Fig. 2c and Fig. 2d). Average correlation coefficients were 0.32, 0.18 and 0.10, mean Jaccard indices were 0.24, 0.08 and 0.14 for MSEA/Mummichog, MSEA/ORA and ORA/Mummichog, respectively.

### Which method delivers consistent results under similar conditions?

**Fig. 3:**
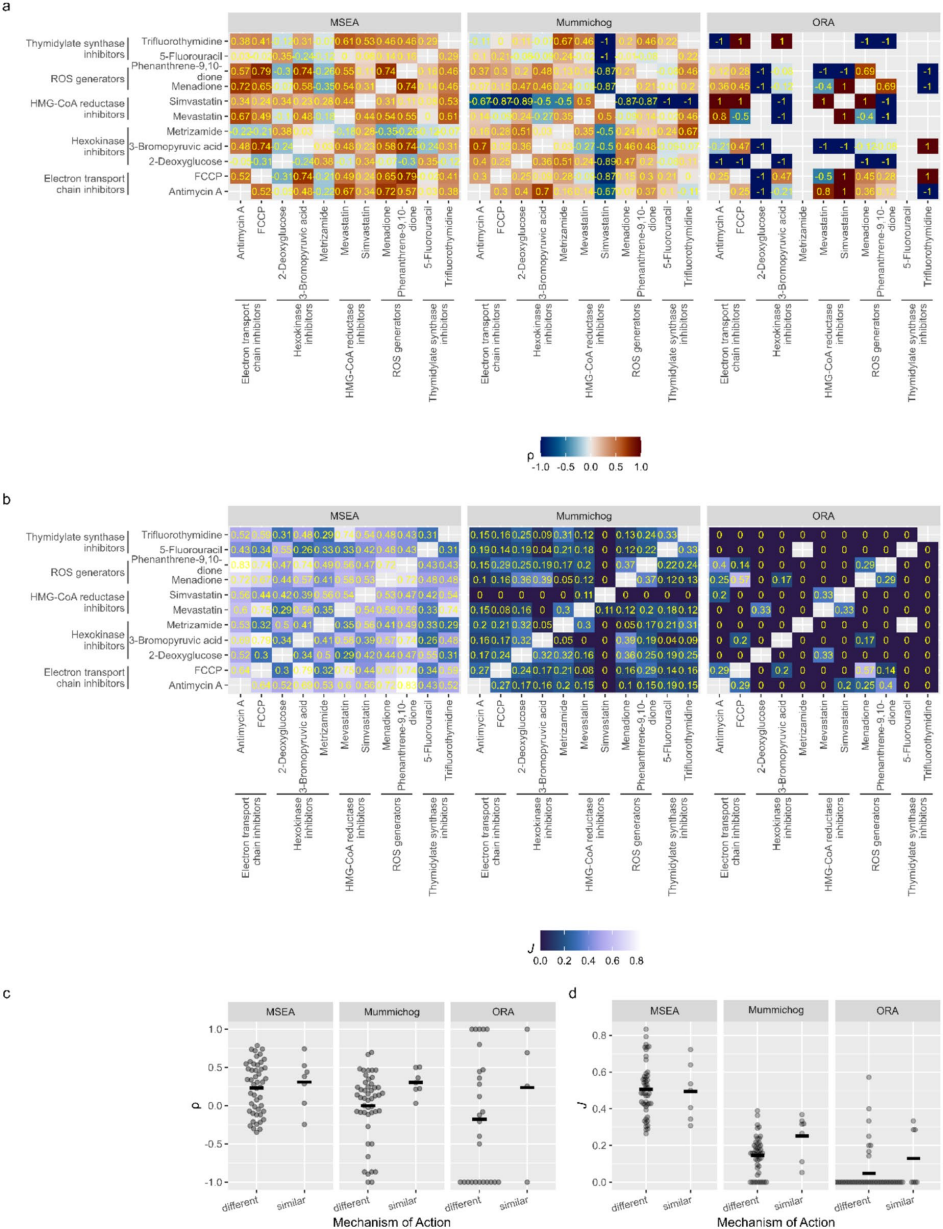
Comparison of the results obtained with the same enrichment method for different compounds. **a** shows the calculated correlation coefficients (Spearman’s ρ), **b** the Jaccard indices *J.* **c** and **d** show the values from **a** and **b** in an alternative way. An emphasis was put on the comparison between compounds with a different mechanism of action to those with a similar mechanism of action. Black vertical lines represent the means

Next, it was investigated which enrichment methods produce consistent results under similar conditions. Similar to the paragraph above, pairwise correlation coefficients and Jaccard indices were calculated, but this time for data sets of different compounds analyzed with the same enrichment method. The idea behind this approach was that both metrics should generally be higher for compounds with a similar MOA compared to compounds with different MOA. The results are summarized in Fig. 3. Mean correlation coefficients were 0.23 and 0.31 for MSEA, 0.00 and 0.31 for Mummichog and -0.18 and 0.24 for ORA for compounds with different and similar MOA, respectively. Mean Jaccard indices were 0.51 and 0.49 for MSEA, 0.15 and 0.25 for Mummichog and 0.05 and 0.13 for ORA for compounds with different and similar MOA, respectively. Figure 3c shows that average correlation coefficients for compounds with similar MOA were similar for all three methods. However, for MSEA the average correlation coefficient for compounds with different MOA was very close, indicating a low specificity of the method. For Mummichog and ORA, the averages lie further apart, with the largest observed difference for ORA. It should, however, be noted that correlation coefficients for ORA have a much larger spread and many could not be calculated due to insufficient data. For Jaccard indices (Fig. 3d), MSEA produced the highest values on average for compounds with a similar MOA, followed by Mummichog and ORA. As with the correlation coefficients, the average Jaccard index for compounds with different MOA was again close to that of compounds with similar MOA for MSEA, indicating a low specificity. The largest difference in averages was observed for Mummichog.

### Which methods deliver correct results?

**Fig. 4:**
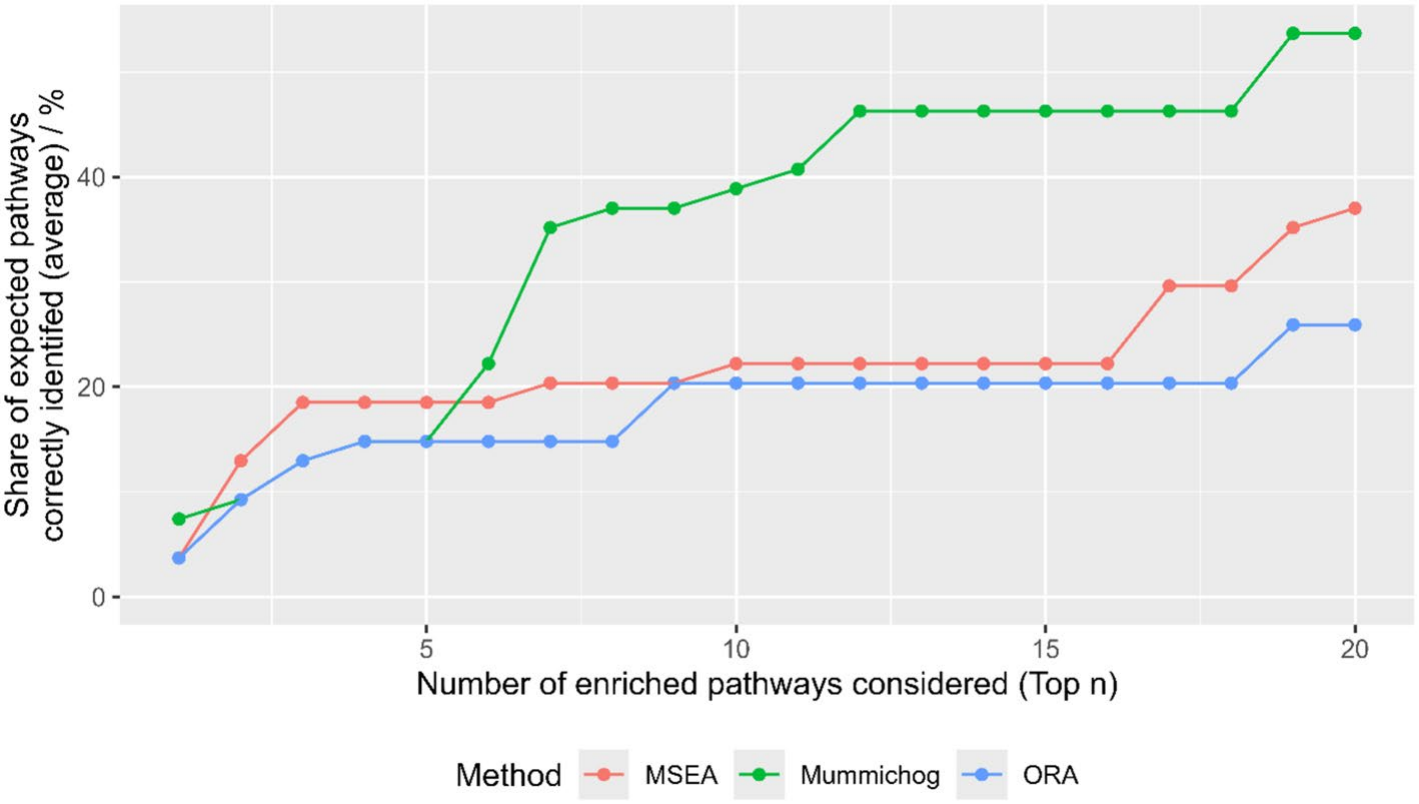
Percentage of expected pathways correctly identified for each enrichment analysis method on average plotted against the number of top enriched pathways included

Probably the hardest question to answer but also the most important one was which methods produce correct results. An intuitive approach, namely comparing the results obtained with each method for each compound to a list of pathways which we would expect for that compound (Supplement S8), was used. The shares of expected pathways correctly identified for each compound were calculated for all three methods. Figure 4 shows the average percentage of correctly identified pathways if the top one to top 20 most significantly enriched pathways are included. It can be seen that Mummichog clearly outperforms both other methods if more than the top five pathways are included. If the top ten pathways are included, Mummichog is able to correctly identify approximately 40% of the expected pathways on average. The performance of ORA is comparable to that of MSEA.

## Discussion

Enrichment analysis has gained large interest in the metabolomics community over the last years, which is reflected by the enormous number of daily users who use the method on MetaboAnalyst (Lu et al., 2023). In vitro testing is a pillar of modern toxicology as well as pharmacology testing, especially considering that the 3R principle—Replace, Reduce, Refine—practically dictates a shift away from animal methods (Gorzalczany & Rodriguez Basso, 2021; Schmeisser et al., 2023). In this work, the three widely used enrichment analysis methods MSEA, Mummichog and ORA were compared using data sets obtained by treating Hep-G2 cells with several pharmacologically and toxicologically relevant compounds with a wide range of MOA. The results obtained with each method were compared and the consistency and correctness of the individual methods was assessed.

A low to moderate similarity between the individual enrichment analysis methods was observed. This discrepancy might be explained by the different statistical approaches of the methods and is in line with findings published by Lu et al. (2023) for data from in vivo studies.

To assess which methods produce consistent results, results for cells treated with different compounds and data analyzed with the same enrichment analysis method were compared. Some similarity in the enrichment analysis results is expected also for compounds with different MOA. While we aimed to cover a diverse range of MOA, some compounds with different primary targets might result in similar pathways being perturbed, either through off-target or through downstream effects. Vice versa, compounds with similar MOA might also interfere with distinct off-targets which might lead to differences in the metabolic changes they cause. For example, besides inhibiting the hexokinase, 2-deoxyglucose also interferes with certain post-translational protein modifications and triggers endoplasmic reticulum stress response, while 3-bromopyruvic acid also acts as an alkylating agent and inhibits the isocitrate dehydrogenase, α-ketoglutarate dehydrogenase and succinate dehydrogenase, possibly resulting in slightly different metabolic profiles (Pelicano et al., 2006; Tziortzioti, 2016). However, similarity for compounds with similar MOA should, in general, be higher than for compounds with different MOA even though the changes in metabolic profiles the individual compounds cause might be slightly different. Of the three methods, Mummichog performed best, considering both metrics used in this publication (Jaccard indices and correlation coefficients). ORA also yielded higher correlation coefficients and Jaccard indices on average for compounds with similar MOA compared to those with different MOA. However, in many cases ORA did not provide enough pathways to calculate a correlation coefficient or a meaningful Jaccard index. MSEA showed the worst performance. Here, the average correlation coefficient and Jaccard index was practically the same for compounds with similar MOA compared to those with different MOA.

In terms of correctness, Mummichog outperformed both other methods. The good overall performance was concordant with the results from Lu et al. (2023). Here, Mummichog was also the best-performing method for data sets from in vivo studies. There are several limitations concerning the assessment of correctness used in this work. First, there is a strong interconnectedness in the metabolome. For example, the inhibition of the electron transport chain and a subsequent decrease in ATP might lead to compensatory up-regulation of alternative pathways for ATP generation, like glycolysis (Ashton et al., 2018; Y. Yang et al., 2020). A relatively short incubation time of 2 h was selected to keep the influence of compensatory effects small. However, some reactions might be faster than that (Wegner et al., 2015). Second, perturbation of one pathway might have effects on downstream pathways. Third, there might be pathway perturbations stemming from potential off-target effects discussed in the section above. All these effects are hard, if not impossible to predict and associated pathways could therefore not be considered in the lists of expected pathways. Finally, all three enrichment analysis methods were used mostly with default parameters including default KEGG pathway libraries (Supplement S7). The reasoning was that it is most likely that the majority of researchers will not bring their own pathway library and will instead use one of the defaults available on MetaboAnalyst 6.0 (Pang et al., 2024). The consequence is that the pathway library used for MSEA and ORA was slightly different from the one used for Mummichog. While they are all based on the KEGG pathway database (Kanehisa et al., 2025) some pathway definitions might vary. It has been shown that the choice of the pathway database and especially the sizes of the pathway definitions can have an impact on the results of enrichment analyses (Karp et al., 2021; Mubeen et al., 2022).

This work comes with several limitations. Only one data pretreatment pipeline was used prior to enrichment analysis. While a comparison of different scaling and normalization techniques was beyond the scope of this work, it has long been known that they can significantly alter the analysis outcome (Van Den Berg et al., 2006). Spectral library search was used as means to annotate metabolites for MSEA and ORA. While this method is convenient and straight forward, especially considering the huge and readily available MS/MS libraries like the ones available from the Global Natural Product Social Molecular Networking (GNPS) (Wang et al., 2016), other approaches for metabolite annotation have been demonstrated to also yield good results (Chen et al., 2021; Lu et al., 2023) The fact that for MSEA and ORA only metabolites validated by MS/MS spectrum were included (i.e., those with a confidence level 2 (Schymanski et al., 2014)) might, at least partly, explain why Mummichog performed better than MSEA and ORA. The prior uses *m/z* as input which is much more data rich. Additionally, confidence in enrichment analysis results could be improved by using an in-house library of authentic standards for metabolite annotation, rather than generic spectral libraries (i.e., elevating the confidence level to 1 (Schymanski et al., 2014)). Finally, unannotated data was used as input for the Mummichog algorithm. Semi-annotating the data might further improve the performance of this algorithm (Lu et al., 2023).

In this study, cells used for metabolome analysis were cultured in 60 mm dishes. This format is far from ideal in terms of throughput, material consumption and amount of compound needed for cell treatment. Future works should investigate whether the good performance of Mummichog still holds for smaller formats where the amounts of metabolites are lower, and measurement uncertainties are expected to be higher.

## Supplementary Information

Below is the link to the electronic supplementary material.

Supplementary file1 (DOCX 288 KB)

Supplementary file 2 (ZIP 72 KB)

## Data Availability

The metabolomics and metadata reported in this paper are available via MetaboLights (Yurekten et al., 2024) https://www.ebi.ac.uk/metabolights/MTBLS12332 study identifier MTBLS12332.

## Code availability

The R code to reproduce the findings reported in this paper is available via GitHub (https://github.com/yasche/Pathway_comparison_SI).
