# Supplementary material for: Comparison of popular enrichment methods for untargeted in vitro metabolomics
Source: Metabolomics. 2025 Jul 27;21(4):103. doi: 10.1007/s11306-025-02309-0 (PMC12301278; doi:10.1007/s11306-025-02309-0)
Supplement: Supplementary file 1 — Supplementary file1 (DOCX 288 KB) [file 11306_2025_2309_MOESM1_ESM.docx]

Supplemental Information

S1 Chemical structures of the compounds used for treatment


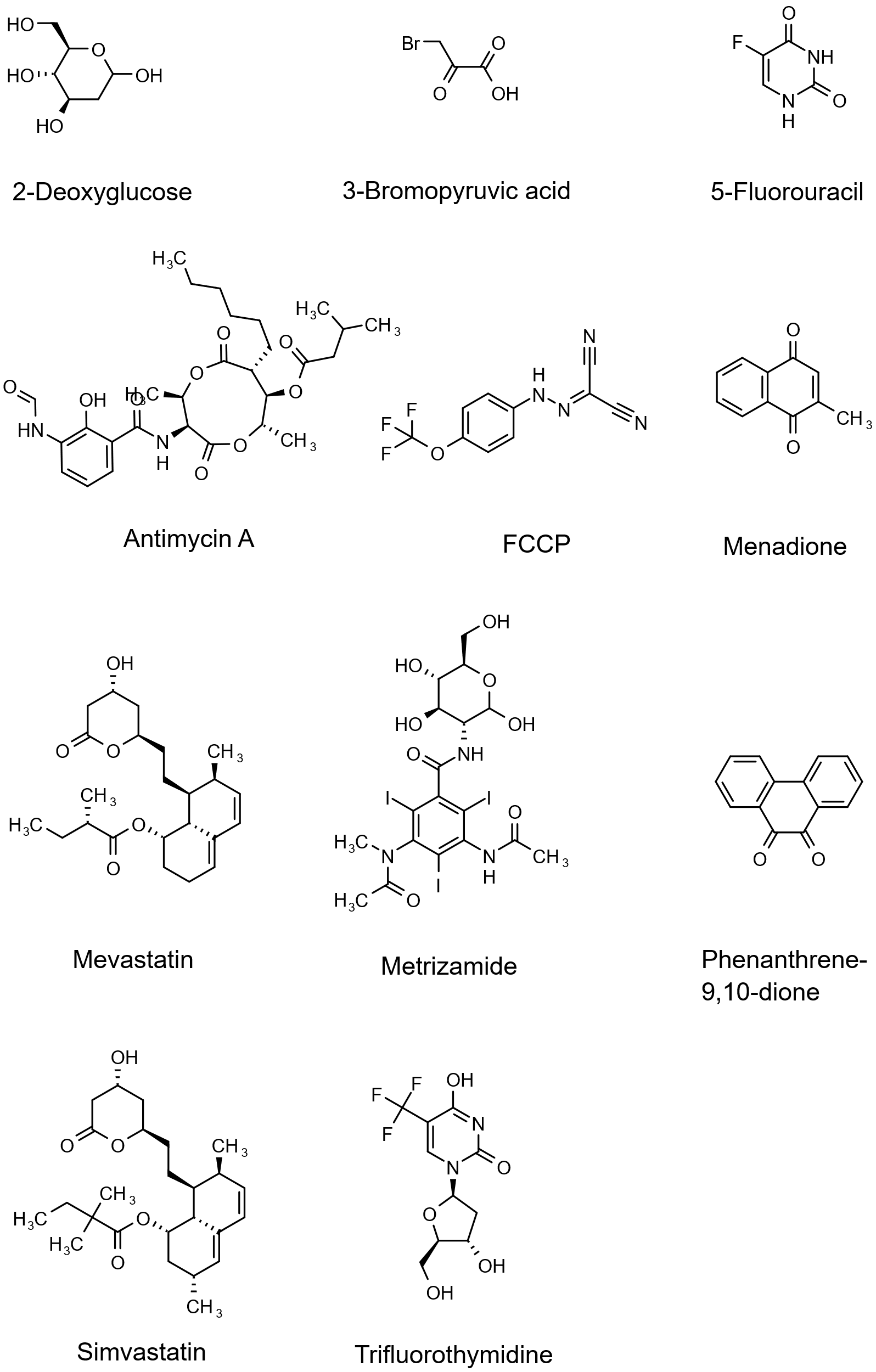


S2 Compound concentrations used for cell treatment

| **Compound** | **Concentration / µmol ⋅ l^-1^** |
| --- | --- |
| Antimycin A | 94.4 |
| 3-Bromopyruvic acid | 78.9 |
| FCCP | 53.4 |
| Menadione | 36.8 |
| Mevastatin | 77.5 |
| Phenanthrene-9,10-dione | 24.8 |
| Simvastatin | 43.0 |
| 2-Deoxyglucose | 1000.0 |
| 5-Fluorouracil | 1000.0 |
| Metrizamide | 1000.0 |
| Trifluorothymidine | 1000.0 |

S3 Gradient table for the reversed phase liquid chromatography method

| **Time / min** | **% A  (water, 0.1 % formic acid)** | **% B  (acetonitrile, 0.1 % formic acid)** | **Flow / µl ⋅ min^-1^** |
| --- | --- | --- | --- |
| 0.0 | 95 | 5 | 300 |
| 1.0 | 95 | 5 | 300 |
| 7.5 | 0 | 100 | 300 |
| 10.0 | 0 | 100 | 300 |
| 10.1 | 95 | 5 | 300 |
| 15.0 | 95 | 5 | 300 |

S4 Gradient table for the hydrophilic interaction chromatography liquid chromatography method

| **Time / min** | **% A  (90 % water, 10 % acetonitrile, 10 mM ammonium acetate, pH 9)** | **% B  (90 % acetonitrile, 10 % water, 10 mM ammonium acetate, pH 9)** | **Flow / µl ⋅ min^-1^** |
| --- | --- | --- | --- |
| 0.0 | 1 | 99 | 400 |
| 0.5 | 1 | 99 | 400 |
| 6.0 | 40 | 60 | 400 |
| 8.0 | 80 | 20 | 400 |
| 9.0 | 80 | 20 | 400 |
| 10.0 | 1 | 99 | 400 |
| 15.0 | 1 | 99 | 400 |

S5 Mass spectrometry parameters

| **Polarity** | **Parameter** | **Value** |
| --- | --- | --- |
| Both | Mass range | *m/z* 20 – 1300 |
| Both | 1/K_0_ | 0.45 – 1.45 Vs/cm^2^ |
| Both | Ramp time | 100 ms |
| Both | ESI source end plate offset | 500 V |
| Both | Nebulizer | 2.2 bar |
| Both | Dry gas flow | 9 l/min |
| Both | Dry temperature | 220 °C |
| Negative | Capillary voltage | 3600 V |
| Negative | Collision energy | -20 eV & -50 eV |
| Positive | Capillary voltage | 4500 V |
| Positive | Collision energy | 20 eV & 50 eV |

S6 Processing parameters

| **Polarity** | **LC-Method** | **Parameter** | **Value** |
| --- | --- | --- | --- |
| Both | Both | MS2 spectrum import | On |
| Both | Both | Mass range | *m/z* 50 – 1500 |
| Both | HILIC | Crop chromatograms after | 9 min |
| Both | Reversed phase | Crop chromatograms after | 10 min |
| Both | Both | Seed intensity | 1000 |
| Both | Both | Minimum cluster size | 75 |
| Both | Both | Minimum seed cluster size | 100 |
| Both | Both | Chromatographic alignment mobility delta | 0.05 |
| Both | Both | Chromatographic alignment mass delta | *m/z* 0.015 |
| Both | Both | Recursive feature extraction | On |
| Both | Both | Group features mobility threshold | 0.05 |
| Both | Both | Group features mass threshold | *m/z* 0.015 |
| Both | Both | Group features retention time threshold | 10 s |
| Both | Both | Ion deconvolution EIC | 0.8 |
| Negative | HILIC | Ion deconvolution ions | primary ion [M-H]^-^  common ion [M-H-H2O]^-^  seed ions [M+Cl]^-^[M+CH3CO2]^-^ |
| Negative | Reversed phase | Ion deconvolution ions | primary ion [M-H]^-^  common ion [M-H-H2O]^-^  seed ion [M+Cl]^-^ |
| Positive | HILIC | Ion deconvolution ions | primary ion [M+H]^+^  common ion [M+H-H2O]^+^  seed ions [M+Na]^+^ [M+K]^+^ [M+NH4]^+^ |
| Positive | Reversed phase | Ion deconvolution ions | primary ion [M+H]^+^  common ion [M+H-H2O]^+^  seed ions [M+Na]^+^ [M+K]^+^ |
| Both | Both | Within-batch correction | On, QC samples |
| Both | Both | Within-batch correction algorithm | LOESS, bandwidth = 0.7 |
| Both | Both | Post-correction variance threshold | 20 |
| Both | Both | Pre-correction variance threshold | 40 |
| Negative | Both | Mass recalibration | On, Na formate negative ion list |
| Positive | Both | Mass recalibration | On, Na formate positive ion list |

S7 Enrichment analysis parameters

| **Method** | **Parameter** | **Value** |
| --- | --- | --- |
| MSEA/ORA | Input/ID type | KEGG ID |
| MSEA/ORA | Feature type | Metabolites |
| MSEA/ORA | Metabolite set | KEGG |
| All | Minimum entries per metabolite set | 2 |
| MSEA | Group label | Categorial |
| MSEA | Filtering | None |
| MSEA | Normalization | None |
| MSEA | Reference metabolome | None |
| Mummichog | Ion mode | Mixed |
| Mummichog | Mass tolerance | 5.0 ppm |
| Mummichog | Retention time | Not present |
| Mummichog | Ranked by | *p*-values |
| Mummichog | Enforce primary ions | Yes |
| Mummichog | Algorithm | Mummichog |
| Mummichog | *p*-value cutoff | Default top 10 % peaks |
| Mummichog | Pathway Library | Homo sapiens, KEGG |

S8 Target-associated pathways. NA: Not Available.

| **Compound** | **Target/MOA** | **Target-associated pathways** | **Pathway ID** |
| --- | --- | --- | --- |
| 2-Deoxyglucose | Hexokinase | Glycolysis / Gluconeogenesis  Fructose and mannose metabolism  Galactose metabolism  Starch and sucrose metabolism  Amino sugar and nucleotide sugar metabolism  Neomycin, kanamycin and gentamicin biosynthesis | hsa00010  hsa00051  hsa00052  hsa00500  hsa00520  hsa00524 |
| 3-Bromopyruvic acid | Hexokinase | Glycolysis / Gluconeogenesis  Fructose and mannose metabolism  Galactose metabolism  Starch and sucrose metabolism  Amino sugar and nucleotide sugar metabolism  Neomycin, kanamycin and gentamicin biosynthesis | hsa00010  hsa00051  hsa00052  hsa00500  hsa00520  hsa00524 |
| 5-Fluorouracil | Thymidylate Synthase | Pyrimidine metabolism  One carbon pool by folate | hsa00240  hsa00670 |
| Antimycin A | Complex III | NA | NA |
| FCCP | Mitochondrial decoupler, indirect inhibition of F-type ATP-synthase | NA | NA |
| Menadione | ROS formation | Cysteine and methionine metabolism  Glutathione metabolism | hsa00270  hsa00480 |
| Metrizamide | Hexokinase | Glycolysis / Gluconeogenesis  Fructose and mannose metabolism  Galactose metabolism  Starch and sucrose metabolism  Amino sugar and nucleotide sugar metabolism  Neomycin, kanamycin and gentamicin biosynthesis | hsa00010  hsa00051  hsa00052  hsa00500  hsa00520  hsa00524 |
| Mevastatin | HMG-CoA Reductase | Terpenoid backbone biosynthesis | hsa00900 |
| Phenanthrene-9,10-dione | ROS formation | Cysteine and methionine metabolism  Glutathione metabolism | hsa00270  hsa00480 |
| Simvastatin | HMG-CoA Reductase | Terpenoid backbone biosynthesis | hsa00900 |
| Trifluorothymidine | Thymidylate Synthase | Pyrimidine metabolism  One carbon pool by folate | hsa00240  hsa00670 |
